# *Aspergillus* Tracheobronchitis Causing Subtotal Tracheal Stenosis in a Liver Transplant Recipient

**DOI:** 10.1155/2013/928289

**Published:** 2013-07-25

**Authors:** Sonia Radunz, Carmen Kirchner, Jürgen Treckmann, Andreas Paul, Fuat H. Saner

**Affiliations:** Department of General, Visceral, and Transplantation Surgery, Essen University Hospital, 45122 Essen, Germany

## Abstract

Invasive aspergillosis is recognized as one of the most significant opportunistic infections after liver transplantation. Diagnosis of invasive aspergillosis in transplant recipients has been proven to be challenging, and optimal approach to the treatment of invasive aspergillosis is still controversial. We here present an unusual case of *Aspergillus* tracheobronchitis in the setting of liver transplantation. A 47-year-old female patient with persistent dry cough after liver transplantation developed respiratory insufficiency and was readmitted to the intensive care unit 55 days after liver transplantation. A CT scan revealed subtotal tracheal stenosis; bronchoscopy was performed, and extended white mucus coverings causative of the tracheal stenosis were removed. Microbiological assessment isolated *Aspergillus fumigatus*. The diagnosis was obstructive *Aspergillus* tracheobronchitis. The patient was started on a treatment of voriconazole 200 mg orally twice daily, adjusted to a trough level of 1–4 mg/L. For further airway management, a tracheal stent had to be implanted. The patient is alive and well 28 months after liver transplantation. Invasive aspergillosis should be considered a possible etiology in liver transplant patients presenting with unspecific symptoms such as persistent dry cough. Optimal strategies for improved and early diagnosis as well as prophylaxis need to be defined.

## 1. Introduction

Invasive aspergillosis is one of the most significant opportunistic infections in solid-organ transplant recipients, and its diagnosis carries a high mortality rate [1]. Early diagnosis of invasive aspergillosis has been proven to be challenging, and the optimal approach to the treatment of invasive aspergillosis is still controversial.

*Aspergillus* tracheobronchitis is a rare but severe form of invasive pulmonary aspergillosis in which the infection is entirely or predominantly confined to the tracheobronchial tree. Up to now, approximately 150 cases have been published in the English literature since 1985 [2]. Approximately 75% of patients with *Aspergillus* tracheobronchitis are immunocompromised. Of the reported cases, approximately 45% were solid-organ transplant recipients with a median time between transplantation and symptom onset of three months. Initial symptoms are generally deceptively mild. Patients often present with nonspecific respiratory symptoms for example, cough, dyspnea, stridor, or wheezing, and radiographic images frequently reveal no relevant findings. Delay of diagnosis and delayed initiation of targeted treatment remain critical for patient outcome. Approximately 30% of patients develop acute respiratory distress. Overall hospital mortality is approximately 40%.

Denning proposed a classification and a unified terminology consisting of three types of *Aspergillus* tracheobronchitis [3]. Ulcerative *Aspergillus* tracheobronchitis is characterized by focal, ulcerative processes with histological invasion of *Aspergillus* species. Pseudomembranous *Aspergillus* tracheobronchitis is characterized by a membrane containing *Aspergillus* species overlaying the mucosa of the entire tracheobronchial tree. Obstructive *Aspergillus* tracheobronchitis is characterized by thick mucous plugs containing *Aspergillus* species without relevant bronchial inflammation.

We describe here the case of a 47-year-old female patient suffering from persistent dry cough 40 days after liver transplantation. To our knowledge, this is the first report of *Aspergillus* tracheobronchitis in a liver transplant recipient, although random cases of *Aspergillus* tracheobronchitis in thoracic organ recipients and hematopoietic stem cell recipients have previously been reported.

## 2. Case Presentation

A 47-year-old female patient was admitted to our department with acute-on-chronic liver failure in the setting of chronic hepatitis B and autoimmune hepatitis. Her past medical history included arterial hypertension and insulin-dependent diabetes mellitus type 2. Initially, she received supportive treatment for liver failure. Due to progressive liver failure resulting in a lab-MELD score of 32, she underwent orthotopic liver transplantation ten days after admission to the hospital. Because of primary nonfunction of the graft, the patient underwent retransplantation within two days.

Immunosuppression consisted of prednisolone 10 mg/kg intraoperatively. Postoperatively, the patient received triple immunosuppression, consisting of tacrolimus 0.1 mg/kg adjusted to a trough level of 8–10 ng/mL, prednisolone 20 mg tapered and withdrawn within six weeks, and mycophenolate mofetil 1 g orally twice daily. For prophylaxis of cytomegalovirus infection, valganciclovir was administered.

The postoperative course was complicated by dialysis-dependent acute renal failure in the setting of initial primary nonfunction of the graft. Due to expected long-term artificial ventilation, tracheotomy was performed on postoperative day (POD) 4. In the further clinical course, kidney function recovered, and dialysis treatment could be stopped. Twelve days after retransplantation, the patient was decannulated and four days later she was transferred to a surgical ward in good clinical condition.

The further postoperative course was unremarkable, except for a persistent dry cough. Regularly performed auscultation did not reveal any abnormal findings; especially no wheezing was detected. A chest X-ray showed no pathological results. Laboratory assessments revealed leucopenia, which was judged to be a side effect of mycophenolate mofetil. Mild laryngitis was diagnosed, and symptomatic treatment with dexpanthenol inhalation was started.

**Figure 1:**
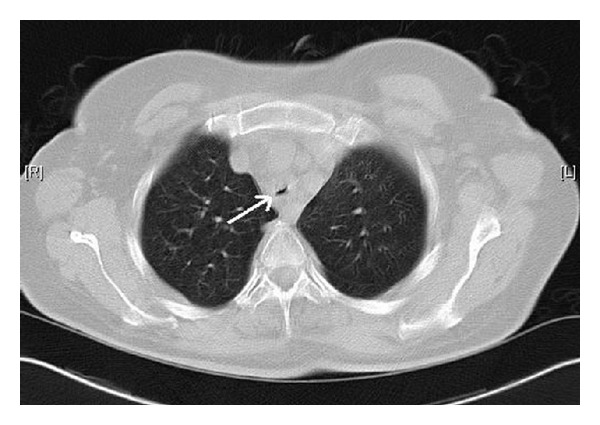
CT scan revealing tracheal stenosis.

**Figure 2:**
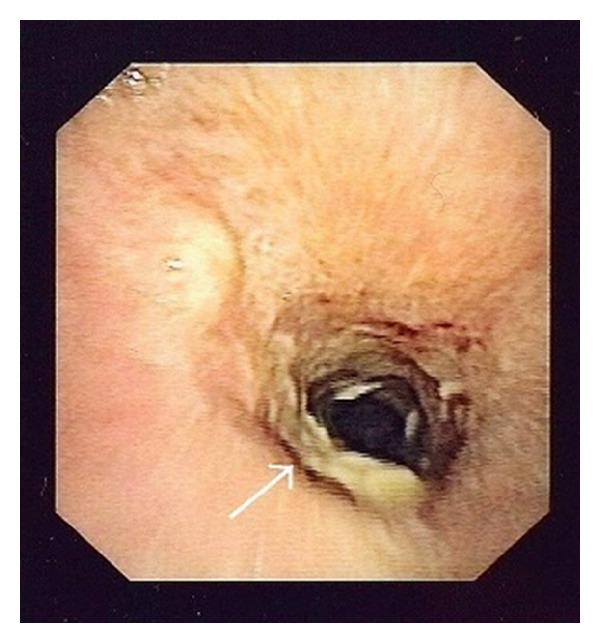
Bronchoscopy revealing extended white mucus coverings causative of the tracheal stenosis.

On POD 55, the patient developed respiratory insufficiency and was readmitted to the intensive care unit. Tracheal stenosis was diagnosed by CT scan (Figure 1). Emergency bronchoscopy was performed, and extended, thick, and white mucous coverings causative of the tracheal stenosis were removed (Figure 2). There were no signs of relevant bronchial inflammation. Microbiological assessments of the removed mucous plugs isolated *Aspergillus fumigatus*. The serum galactomannan index was <0.5. The diagnosis was obstructive *Aspergillus* tracheobronchitis.

The patient was started on a treatment of voriconazole 200 mg orally twice daily, adjusted to a trough level of 1–4 mg/L. Voriconazole was maintained for five months. In the setting of severe infection, mycophenolate mofetil was paused, and tacrolimus dosage was adjusted accordingly because of drug interaction to a trough level of 5–7 ng/mL [4].

**Figure 3:**
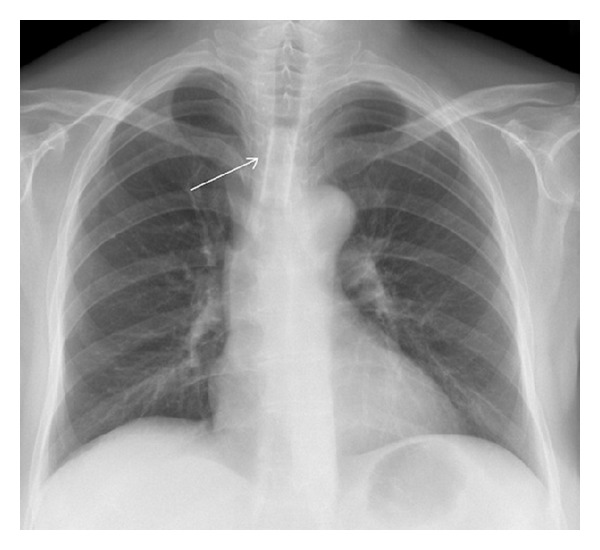
Chest X-ray with tracheal stent in place.

The persistent dry cough subsided, and a follow-up bronchoscopy on POD 59 showed a clearance of the extended white mucus coverings. On POD 71, an emergency bronchoscopy had to be performed again due to inspiratory stridor. For further airway management, a tracheal stent was implanted because of residual tracheal stenosis (Figure 3). On POD 80, the patient was discharged in good clinical condition. Twenty-eight months after liver transplantation, the patient is alive and well. The tracheal stent was removed after five months. For follow-up, the patient is seen in our outpatient clinic. The patient is routinely treated endoscopically for ischemic-type biliary lesion.

## 3. Discussion

Infections are a common cause of morbidity and mortality in solid-organ transplant recipients. Invasive aspergillosis is recognized as one of the most significant opportunistic infections in these patients. *Aspergillus spp.* have been isolated from approximately 9%–34% of patients with invasive fungal infection after liver transplantation [1, 5]. Risk factors for invasive aspergillosis in liver transplant recipients include pretransplant hepatic failure, primary graft dysfunction or nonfunction, retransplantation, dialysis treatment, and high transfusion requirement [6].

*Aspergillus* tracheobronchitis is a rare finding with deceptively mild symptoms and absence of radiographic abnormalities; therefore, early diagnosis is challenging. The reported incidence is much higher in lung transplant recipients, and so far no cases have been reported after liver, pancreas, or intestine transplantation [2, 7].

Besides a persistent dry cough, there were no findings suspicious of any infection in our patient. The differential diagnosis of persistent dry cough is broad and includes upper respiratory tract infection (“common cold”), allergic asthma, psychogenic cough, drug side effects (ACE inhibitors), (atypical) pneumonia, and lung cancer. In our patient, possible causative allergens were eliminated, and there were no clinical signs of infection or malignancy. Therefore, simply symptomatic treatment was initiated. A final diagnosis of invasive aspergillosis was only possible when the patient became severely symptomatic, and microbiological assessment of samples collected during bronchoscopy revealed *Aspergillus fumigatus*.

The galactomannan test failed to demonstrate invasive aspergillosis in our patient. The definitive role of the galactomannan test in early diagnosis of invasive aspergillosis without lung parenchyma involvement still has to be established. The galactomannan test was evaluated in hematopoietic stem cell patients with conflicting results [8–12]. In a study with lung transplant patients, the test detected only 29% of patients with invasive aspergillosis and failed to detect patients with *Aspergillus* tracheobronchitis [13]. A subsequent study in lung transplant patients indicated that a higher cutoff for the galactomannan antigen for aspergillosis of 1 in the bronchoalveolar lavage increases the specificity to 98% [14].

An optimal approach to the treatment of invasive aspergillosis is still controversial. Voriconazole is now regarded as the drug of choice for primary treatment of invasive aspergillosis, a recommendation endorsed by the Infectious Diseases Society of America (IDSA) for the treatment of invasive aspergillosis [15]. In a prospective randomized trial, the successful outcome and survival rate in the voriconazole group were significantly higher as compared with the amphotericin B deoxycholate group. Also, voriconazole-treated patients had fewer side effects, except for transient visual disturbances [16]. Caspofungin is currently the only echinocandin which is approved by the FDA for treatment of aspergillosis. Caspofungin was successfully used as first-line therapy in heart-lung transplant patients and as salvage therapy in invasive aspergillosis as a single agent [17, 18]. Posaconazole, a new extended-spectrum triazole, was successfully applied as rescue treatment for patients refractory or intolerant to conventional therapy in a prospective open-label study [19].

Increasingly, antifungal combination therapy is used despite limited data existence with regard to the clinical efficacy of combination therapy for invasive aspergillosis. Perea et al. investigated the *in vitro* interaction of caspofungin and voriconazole and their results indicated that a combination of caspofungin and voriconazole might be more effective against infections caused by *Aspergillus spp.* as compared with single treatment [20]. In a clinical trial with solid-organ transplant recipients with proven invasive aspergillosis, including liver transplant patients, Singh et al. prospectively assessed a combination therapy consisting of caspofungin and voriconazole in comparison with a historical control group which was treated with liposomal amphotericin B [21]. The overall 90-day survival rate was not different in both groups, but patients infected with *Aspergillus fumigatus* and patients with renal failure showed a significant better 90-day survival. Therefore, a combination of voriconazole and caspofungin might be considered as preferable therapy for certain subsets of organ transplant recipients with invasive aspergillosis. The latest IDSA guidelines recommend combination therapy as salvage treatment only [22].

Initial antifungal therapy in our patient included voriconazole alone. Reduction in immunosuppression and maintenance therapy with voriconazole as well as interventional therapy by tracheal stenting resulted in a favorable outcome. Final follow-up bronchoscopy and follow-up CT scan showed a significant decrease of the tracheal stenosis. 

In conclusion, we need to be aware of *Aspergillus* tracheobronchitis as a potential diagnosis in transplant recipients and other immunocompromised patients with nonspecific respiratory symptoms and few radiographic abnormalities. Future studies should address optimal strategies for improved and early diagnosis as well as standardized treatment. Anti-fungal prophylaxis in high-risk liver transplant recipients may be warranted.

## References

[1] Gabardi S, Kubiak DW, Chandraker AK, Tullius SG (2007). Invasive fungal infections and antifungal therapies in solid organ transplant recipients. *Transplant International*.

[2] Fernandez-Ruiz M, Silva JT, San-Juan R (2012). *Aspergillus* tracheobronchitis: report of 8 cases and review of the literature. *Medicine*.

[3] Denning DW (1995). Commentary: unusual manifestations of aspergillosis. *Thorax*.

[4] Venkataramanan R, Zang S, Gayowski T, Singh N (2002). Voriconazole inhibition of the metabolism of tacrolimus in a liver transplant recipient and in human liver microsomes. *Antimicrobial Agents and Chemotherapy*.

[5] Pacholczyk MJ, Lagiewska B, Lisik W, Wasiak D, Chmura A (2011). Invasive fungal infections following liver transplantation—risk factors, incidence and outcome. *Annals of Transplantation*.

[6] Rosenhagen M, Feldhues R, Schmidt J, Hoppe-Tichy T, Geiss HK (2009). A risk profile for invasive aspergillosis in liver transplant recipients. *Infection*.

[7] Ramos A, Segovia J, Gómez-Bueno M (2010). Pseudomembranous aspergillus tracheobronchitis in a heart transplant recipient: case report. *Transplant Infectious Disease*.

[8] Herbrecht R, Letscher-Bru V, Oprea C (2002). *Aspergillus* galactomannan detection in the diagnosis of invasive aspergillosis in cancer patients. *Journal of Clinical Oncology*.

[9] Maertens J, Theunissen K, Verbeken E (2004). Prospective clinical evaluation of lower cut-offs for galactomannan detection in adult neutropenic cancer patients and haematological stem cell transplant recipients. *British Journal of Haematology*.

[10] Maertens J, Theunissen K, Verhoef G, Van Eldere J (2004). False-positive *Aspergillus* galactomannan antigen test results. *Clinical Infectious Diseases*.

[11] Siemann M, Koch-Dörfler M, Gaude M (1998). False-positive results in premature infants with the Platelia *Aspergillus* sandwich enzyme-linked immunosorbent assay. *Mycoses*.

[12] Verweij PE, Dompeling EC, Donnelly JP, Schattenberg AVMB, Meis JFGM (1997). Serial monitoring of *Aspergillus* antigen in the early diagnosis of invasive aspergillosis. Preliminary investigations with two examples. *Infection*.

[13] Husain S, Kwak EJ, Obman A (2004). Prospective assessment of Platelia*™Aspergillus* galactomannan antigen for the diagnosis of invasive aspergillosis in lung transplant recipients. *American Journal of Transplantation*.

[14] Husain S, Paterson DL, Studer SM (2007). *Aspergillus* galactomannan antigen in the bronchoalveolar lavage fluid for the diagnosis of invasive aspergillosis in lung transplant recipients. *Transplantation*.

[15] Pappas PG, Kauffman CA, Andes D (2009). Clinical practice guidelines for the management of candidiasis: 2009 update by the Infectious Diseases Society of America. *Clinical Infectious Diseases*.

[16] Herbrecht R, Denning DW, Patterson TF (2002). Voriconazole versus amphotericin B for primary therapy of invasive aspergillosis. *The New England Journal of Medicine*.

[17] Groetzner J, Kaczmarek I, Wittwer T (2008). Caspofungin as first-line therapy for the treatment of invasive aspergillosis after thoracic organ transplantation. *Journal of Heart and Lung Transplantation*.

[18] Carby MR, Hodson ME, Banner NR (2004). Refractory pulmonary aspergillosis treated with caspofungin after heart-lung transplantation. *Transplant International*.

[19] Walsh TJ, Raad I, Patterson TF (2007). Treatment of invasive aspergillosis with posaconazole in patients who are refractory to or intolerant of conventional therapy: an externally controlled trial. *Clinical Infectious Diseases*.

[20] Perea S, Gonzalez G, Fothergill AW, Kirkpatrick WR, Rinaldi MG, Patterson TF (2002). In vitro interaction of caspofungin acetate with voriconazole against clinical isolates of *Aspergillus* spp. *Antimicrobial Agents and Chemotherapy*.

[21] Singh N, Limaye AP, Forrest G (2006). Combination of voriconazole and caspofungin as primary therapy for invasive aspergillosis in solid organ transplant recipients: a prospective, multicenter, observational study. *Transplantation*.

[22] Walsh TJ, Anaissie EJ, Denning DW (2008). Treatment of aspergillosis: clinical practice guidelines of the infectious diseases society of America. *Clinical Infectious Diseases*.

